# *Quo vadis?* Central Rules of Pathogen and Disease Tropism

**DOI:** 10.3389/fcimb.2021.640987

**Published:** 2021-02-25

**Authors:** Laura-Isobel McCall

**Affiliations:** ^1^ Department of Chemistry and Biochemistry, University of Oklahoma, Norman, OK, United States; ^2^ Department of Microbiology and Plant Biology, University of Oklahoma, Norman, OK, United States; ^3^ Stephenson Cancer Center, University of Oklahoma, Oklahoma City, OK, United States; ^4^ Laboratories of Molecular Anthropology and Microbiome Research, University of Oklahoma, Norman, OK, United States

**Keywords:** disease tropism, pathogen tropism, bacteria, viruses, fungi, parasites, treatment

## Abstract

Understanding why certain people get sick and die while others recover or never become ill is a fundamental question in biomedical research. A key determinant of this process is pathogen and disease tropism: the locations that become infected (pathogen tropism), and the locations that become damaged (disease tropism). Identifying the factors that regulate tropism is essential to understand disease processes, but also to drive the development of new interventions. This review intersects research from across infectious diseases to define the central mediators of disease and pathogen tropism. This review also highlights methods of study, and translational implications. Overall, tropism is a central but under-appreciated aspect of infection pathogenesis which should be at the forefront when considering the development of new methods of intervention.

## Introduction

*Why* disease and death occur are intrinsically tied to *where* they happen, referred to as tropism. Pathogen tropism describes the locations that can become infected by a given infectious agent (bacterial, viral, fungal or parasitic), while disease tropism is the location of the resulting damage, impairing healthy function. In this context, we will not consider host tropism (the host range that can be infected by a pathogen), focusing instead on human pathogens and animal models thereof (**Table 1**). Tropism can be defined from the smallest subcellular scale to the broadest geographic scales, and is influenced by pathogen factors, intrinsic host characteristics such as immune status or genetic background, and external factors such as climate. Tropism is a leading determinant of disease severity (Brierley et al., 2019). Understanding tropism has been a central aspect of research efforts on newly-emerging or re-emerging pathogens such as Zika virus (Ma et al., 2016; Miner et al., 2016) or SARS-CoV-2 (Trypsteen et al., 2020; Ziegler et al., 2020), for example. For pathogens which have a longer history of human infectivity, studying tropism is not only leading to an improved understanding of pathogenesis mechanisms, but may also help guide the development of the next generation of rational therapeutics for infectious diseases.

**Table 1:** Pathogens discussed.

Viruses				
Name	Disease	Disease tropism and pathognomonic symptoms	Dominant pathogen cellular tropism	Dominant pathogen tissue tropism
Dengue virus	Dengue	Fever, vomiting and bleeding, rash, muscle, joint and bone pain	Macrophages, dendritic cells (Balsitis et al., 2009)	Lymph nodes and spleen; lung, central nervous system (cerebrum), liver (Balsitis et al., 2009)
Epstein–Barr virus (EBV)	Infectious mononucleosis	Fever, sore throat, rash, hepatosplenomegaly	Epithelial cells, B cells (Borza and Hutt-Fletcher, 2002; Shannon-Lowe et al., 2006)	Oropharyngeal epithelium (Shannon-Lowe et al., 2006)
Human Cytomegalovirus (HCMV)	HCMV infection	Usually asymptomatic in immunocompetent individuals; congenital damage; various manifestations in immunocompromised adults	Broad tropism, including epithelial and endothelial cells, leukocytes, smooth muscle and hepatocytes (Scrivano et al., 2011; Almanan et al., 2017)	Broad (Scrivano et al., 2011)
Hepatitis B virus (HBV)	Hepatitis	Liver damage, liver cirrhosis, liver cancer	Hepatocytes (Tang and McLachlan, 2001)	Liver (Tang and McLachlan, 2001)
Hepatitis C virus (HCV)	Hepatitis	Chronic liver disease, liver cirrhosis, liver cancer	Hepatocytes (Neufeldt et al., 2016)	Liver (Neufeldt et al., 2016)
Herpes simplex virus 1 (HSV-1)	Oral and genital herpes	Oral sores	Epithelial cells, neurons (Khoury-Hanold et al., 2016)	Mouth, genitals (Khoury-Hanold et al., 2016)
Herpes simplex virus 2 (HSV-2)	Genital herpes	Genital sores	Epithelial cells, neurons (Khoury-Hanold et al., 2016)	Genitals (Khoury-Hanold et al., 2016)
Human immunodeficiency virus 1 (HIV-1)	AIDS (Acquired immunodeficiency syndrome)	CD4^+^ T cell depletion, opportunistic infections, opportunistic cancers, fever, sweats, wasting, diarrhea	CD4^+^ T cells (Ribeiro et al., 2016)	Multiple locations (Feder et al., 2017; Guzzo et al., 2017)
Influenza virus	influenza (“flu”)	Fever, headache, fatigue, coughing, runny nose, joint and muscle pain	Airway epithelial cells (Scull et al., 2009)	Airways (Scull et al., 2009)
Marburg virus	Hemorrhagic fever	Fever, severe blood loss from multiple sites, inflammation of testicles	Phagocytic cells, Sertoli cells (Coffin et al., 2018)	Multiple locations, including spleen, lymph nodes, liver; persistence in testes (Coffin et al., 2018)
Middle East Respiratory Syndrome Coronavirus (MERS-CoV)	Middle East Respiratory Syndrome (MERS)	Fever, cough, difficulty breathing; sometimes diarrhea or vomiting; complications affecting lung and kidney	Lung epithelial cells (Park et al., 2016)	Lung (Park et al., 2016)
murine cytomegalovirus (MCMV)	Not a human pathogen	Animal model for *Herpesviridae* infection	Broad tropism, including epithelial and endothelial cells, leukocytes, smooth muscle and hepatocytes (Scrivano et al., 2011; Almanan et al., 2017)	Broad (Almanan et al., 2017)
Norovirus	Gastroenteritis	Gastrointestinal symptoms: nausea, vomiting, diarrhea	Intestinal epithelial cells (Lee et al., 2017; Murakami et al., 2020)	Highest in the distal small intestine (Grau et al., 2020)
Poliovirus	Poliomyelitis	Flu-like symptoms in mild infection; severe infection: brain and spinal cord symptoms, up to paralysis	Neurons (Ida-Hosonuma et al., 2005)	Spine, brain stem (Ida-Hosonuma et al., 2005)
Rhinovirus	Common cold	Nasal congestion, sneezing, cough, sore throat; malaise, fever	Airway epithelial cells, fibroblasts, dendritic cells (Foxman et al., 2015)	Nasal cavity; some lower airway infections possible (Foxman et al., 2015)
SARS-CoV-2	COVID-19	Primarily respiratory (coughing, difficulty breathing); also gastrointestinal; fever; complications affecting multiple organs	Lung type II pneumocytes, nasal goblet secretory cells, ileal enterocytes (Ziegler et al., 2020)	Airways (Zhu et al., 2020); extra-respiratory involvement also present (Puelles et al., 2020; Xiao et al., 2020; Ziegler et al., 2020)
Sindbis virus	Sindbis fever	Malaise, joint pain, rash	Epithelial cells, fibroblasts (Varble et al., 2013)	Broad (Ryman et al., 2000; Varble et al., 2013)
Simian immunodeficiency virus (SIV)	Not a human pathogen	Model for HIV infection	CD4^+^ T cells (Stieh et al., 2016)	Broad (Stieh et al., 2016; Feder et al., 2017)
Varicella zoster virus	Varicella, Zoster	Skin lesions	T cells, neurons, fibroblasts (Sen et al., 2014)	Usually skin, nerves (Sen et al., 2014)
West Nile virus	West Nile fever	Usually asymptomatic; fever, fatigue, joint pain, rash, diarrhea, vomiting; central nervous system symptoms in severe cases	Neurons, phagocytic cells (Suthar et al., 2013)	Skin, spleen, central nervous system (Suthar et al., 2013)
Zika virus	Zika virus disease	Often asymptomatic or mild non-specific (fever, muscle pain, rash…); nerve damage (Guillain-Barré syndrome); microcephaly in congenital infection	Neurons, neural progenitors, astrocytes, microglia, Sertoli cells, epithelial cells (Ma et al., 2016; Muffat et al., 2018; Szaba et al., 2018; Hui et al., 2020)	Broad; includes brain, testes, eye, placenta (Hui et al., 2020)
**Bacteria**				
**Name**	**Disease**	**Disease tropism and pathognomonic symptoms**	**Dominant pathogen cellular tropism**	**Dominant pathogen tissue tropism**
*Borrelia burgdorferi*	Lyme disease	Fever, rash, fatigue; can progress to cardiac and central nervous system manifestations and joint pain	Primarily extracellular	Broad (Sertour et al., 2018)
*Campylobacter jejuni*	Campylobacteriosis	Gastrointestinal symptoms: diarrhea	Epithelial cells (Luethy et al., 2017)	Cecum, large intestine (Luethy et al., 2017)
*Chlamydia trachomatis*	Chlamydia	Genital discharge, pain	Epithelial cells (Howe et al., 2019)	Genital organs, lymph nodes, spleen, GI tract (Howe et al., 2019)
*Citrobacter rodentium*	Not a human pathogen	Model for enteropathogenic *Escherichia coli* (EPEC) and enterohaemorrhagic *E. coli* (EHEC)	Extracellular	Large intestine (Thaiss et al., 2018)
*Clostridioides difficile*	“*C. diff”* infection	Gastrointestinal symptoms: diarrhea, stomach pain, nausea; fever	Extracellular	Large intestine (Buffie et al., 2015)
*Coxiella burnetii*	Q fever	Mainly non-specific: fever, aches, malaise, chest or stomach pain, diarrhea, vomiting, cough	Monocytes, macrophages, trophoblasts (Ben Amara et al., 2010)	Broad (Ben Amara et al., 2010)
*Escherichia coli*	food poisoning, urinary tract infections, meningitis (strain-dependent)	Gastrointestinal symptoms: diarrhea, pain, vomiting. Urinary tract symptoms: painful and frequent urination. Meningitis: fever, headache	Extracellular or intracellular (epithelial cells) (Connolly et al., 2015)	Strain-dependent: large intestine, bladder, central nervous system (Connolly et al., 2015; Rajan et al., 2018; Rajan et al., 2020)
*Listeria monocytogenes*	Listeriosis	Fever, diarrhea; in pregnancy: stillbirth, miscarriage, fetal infection; muscle pain, central nervous system manifestations; select localized infections	Epithelial cells (Stavru et al., 2011; Dowd et al., 2020)	Small intestine; liver, spleen, placenta, central nervous system (Pentecost et al., 2006)
*Mycobacterium tuberculosis*	Tuberculosis	Usually respiratory (cough, chest pain); fatigue, weight loss, fever; can also be extrapulmonary	Macrophages (Russell et al., 2019)	Lung (Russell et al., 2019)
*Neisseria gonorrhoeae*	Gonorrhea	Urogenital discharge; pain	Epithelial cells (Roth et al., 2013)	Urogenital tract; can disseminate (Roth et al., 2013)
*Pseudomonas aeruginosa*	*P. aeruginosa* infection	Variable depending on localization (cough for respiratory *P. aeruginosa* infection, discharge for wound infections; fever)	Extracellular	Broad (Bachta et al., 2020)
*Salmonella enterica* serovar Typhimurium	Salmonellosis	Gastrointestinal symptoms: diarrhea, stomach pain, fever	Epithelial cells, macrophages (Knodler et al., 2010; Kurtz et al., 2020)	Intestines, gallbladder, liver (Knodler et al., 2010; Kurtz et al., 2020)
*Shigella*	Shigellosis	Gastrointestinal symptoms: diarrhea, stomach pain, fever	Epithelial cells (Du et al., 2016)	Intestines (Koestler et al., 2019)
*Streptococcus pneumoniae*	Pneumococcal disease	Usually respiratory: pain, cough, shortness of breath; central nervous system infections; bacteremia, otitis	Extracellular	Lung, bloodstream, central nervous system, ear (Minhas et al., 2019)
**Parasites**				
**Name**	**Disease**	**Disease tropism and pathognomonic symptoms**	**Dominant pathogen cellular tropism**	**Dominant pathogen tissue tropism**
*Entamoeba histolytica*	Amoebiasis	Gastrointestinal symptoms: diarrhea, pain; invasion to the liver causing liver abscess can occur	Extracellular	Large intestine, liver (Thibeaux et al., 2014; Siqueira-Neto et al., 2018)
*Leishmania major*	Cutaneous leishmaniasis	Skin lesions	Phagocytes (Zhang et al., 2003; Peters et al., 2008)	Skin (Zhang et al., 2003)
*Leishmania donovani*	Visceral leishmaniasis	Fever, hepatosplenomegaly	Phagocytes (Zhang et al., 2003; Peters et al., 2008)	Liver, spleen, bone marrow (Zhang et al., 2003; McCall et al., 2013)
*Plasmodium falciparum*	Malaria	Fever, chills, anemia, cerebral symptoms	Red blood cells (Pal et al., 2016)	Circulation; sequestration in multiple locations, including brain, spleen, lung, placenta (Brugat et al., 2014; Pal et al., 2016)
*Plasmodium vivax*	Malaria	Fever, chills, anemia, cerebral symptoms	Red blood cells (Brugat et al., 2014)	Circulation; sequestration in multiple locations, including liver, lung, spleen (Brugat et al., 2014)
*Toxoplasma gondii*	Toxoplasmosis	Usually asymptomatic; congenital infections; ocular and central nervous system manifestations	Broad *in vitro*; some *in vivo* cell type preferences, including neurons (Cabral et al., 2016)	Broad (Saeij et al., 2005); persistence in eye, brain
*Trypanosoma brucei*	Sleeping sickness (African trypanosomiasis)	Central nervous system symptoms: behavioral and motor disturbances, coma; fever, malaise	Extracellular	Central nervous system (McCall and McKerrow, 2014)
*Trypanosoma cruzi*	Chagas disease (American trypanosomiasis)	Cardiomyopathy, megacolon, megaesophagus	Broad *in vitro (* Franco et al., 2019 *);* myocytes *in vivo* (Costa et al., 2018)	Persistence primarily in the GI tract; also heart, skin (Lewis et al., 2014; Lewis et al., 2016; Ward et al., 2020)
**Fungi**				
**Name**	**Disease**	**Disease tropism and pathognomonic symptoms**	**Dominant pathogen cellular tropism**	**Dominant pathogen tissue tropism**
*Aspergillus fumigatus*	Aspergillosis	Respiratory: cough, shortness of breath, chest pain; fever; may spread systematically	Extracellular	Lung (Hsu et al., 2018)
**Prions**				
**Name**	**Disease**	**Disease tropism and pathognomonic symptoms**	**Dominant pathogen cellular tropism**	**Dominant pathogen tissue tropism**
Prions (PrP^Sc^)	Transmissible spongiform encephalopathy	Neurological: behavioral and motor abnormalities	Neurons	Central nervous system, lymphoid tissue (Béringue et al., 2020)

## The Spatial Granularity of Tropism

Pathogen tropism within a mammalian host is often an intrinsic characteristic of a given pathogen strain or species, influenced by host characteristics (*e.g.* immune status) (McCall et al., 2013). However, some pathogens are pleiotropic, with variable preferential localizations depending on disease stage: for example. *Trypanosoma cruzi* strain CL Brener is found in all tested visceral organs during acute BALB/c mouse infection. During chronic infection, parasite load is consistently high only in the stomach and colon (Lewis et al., 2014), with the cecum the only site where parasite burden increases during the transition from acute to chronic disease (Hossain et al., 2020). Beyond large-scale tropism to select organs and tissues, pathogens also show finer cellular and subcellular tropism.

### Subcellular Tropism

Intracellular pathogens colonize specific subcellular niches. Some niches may only be transient sites of colonization during pathogen uptake, while others are occupied for most of the pathogen lifecycle. Some pathogens also replicate at multiple subcellular locations. For example, *Salmonella enterica* serovar Typhimurium localize and replicate mainly in the *Salmonella*-containing vacuole. However, a subpopulation of *Salmonella* proliferate in the cytoplasm (Knodler et al., 2010). Viral subcellular tropism enables access to host enzymes essential for productive viral infection and host lipids and proteins to shield the viral particle from the immune system. For example, hepatitis C replicates on viral-induced cytoplasmic structures called the membranous web, which shields the virus from host pattern-recognition receptors (Neufeldt et al., 2016). *Leishmania* parasites and *Coxiella burnetii* bacteria multiply in the phagolysosome, a low-pH environment that requires specific metabolic adaptations for successful colonization (Saunders et al., 2014). In contrast, *T. cruzi* parasites and several bacteria (*e.g. Listeria monocytogenes*) proliferate in the cell’s cytoplasm where nutrients are abundant (Knodler et al., 2010; Lentini et al., 2018). This localization may also facilitate intercellular pathogen transfer (Knodler et al., 2010; Dowd et al., 2020).

Infection can also result in damage at specific subcellular sites. For example, *T. cruzi* causes mitochondrial swelling in chronic Chagas disease (Garg et al., 2003; Gupta et al., 2009). Mitochondrial damage is also observed in Herpes Simplex Virus-1 (HSV-1)-infected myenteric neurons (Khoury-Hanold et al., 2016) and during *L. monocytogenes* epithelial cell infection (Stavru et al., 2011), suggesting that this is a common alteration that may be caused by the metabolic stress of infection, pathogen mechanisms to avoid host cell apoptosis, or avoidance of other host defense mechanisms (Stavru et al., 2011). Examples of other sites of subcellular structural alterations include the endoplasmic reticulum, Golgi and nucleus during dengue virus infection (Win et al., 2019). Subcellular sites of disease tropism may be sites of direct contact with host structures, as in *T. cruzi* infection, in which cytoplasmic parasites interact with the mitochondria *via* parasite flagella (Lentini et al., 2018). In contrast, HSV-1 was distal to damaged mitochondria (Khoury-Hanold et al., 2016).

### Cellular Tropism

Intracellular pathogens preferentially colonize specific cell types. For viruses, this is first driven by the availability of entry receptors, associated processing factors, and fusion mediators, in the appropriate structural conformation (Wang and Shenk, 2005; Park et al., 2016; Orchard et al., 2018) for initial viral entry, or cell-to-cell interactions between infected and uninfected cells leading to viral spread (Shannon-Lowe et al., 2006). Subsequently, cellular tropism is determined by the ability of the infected cell to degrade internalized viruses and/or prevent their proliferation. For example, the poliovirus receptor is expressed on multiple tissues that are not sites of viral replication; viral tropism restriction is due to pre-existing expression of interferon-stimulated genes (ISGs) (Ida-Hosonuma et al., 2005). Similar processes are observed for influenza virus infection across different lung cell types (Fay et al., 2020). Depending on the receptor used for Human Immunodeficiency virus-1 (HIV-1) internalization, host TRIM5α either restricts the virus *via* autophagic targeting (Langerhans cells) or does not (subepithelial DC-SIGN^+^ dendritic cells) (Ribeiro et al., 2016). Last, the availability of the necessary resources to enable viral proliferation also regulates viral tropism. Metabolism controls the building blocks necessary to viral replication (Rodríguez-Sánchez et al., 2019), but recent results have also demonstrated that the availability of metabolites such as ceramide can also regulate virus entry receptor conformation and viral uptake (Orchard et al., 2018).

Apicomplexan parasites actively invade the host cells (Guérin et al., 2017). In contrast, many other intracellular bacterial and eukaryotic pathogens induce their own uptake by the host. For example, *Leishmania* parasites rely on phagocytosis for entry and are thus primarily tropic to neutrophils and then macrophages (Peters et al., 2008). Such host cells may be actively recruited to the sites of initial pathogen colonization by the mechanisms of transmission, for example *via* bioactive proteins and metabolites found in vector saliva or even egested vector gut bacteria (Peters et al., 2008; Dey et al., 2018). Lack of uptake can also determine pathogen tropism: disseminative *Neisseria gonorrhoeae* strains are unable to bind neutrophil receptor CEACAM3, enabling them to avoid immune killing and to disseminate beyond mucosal sites (Roth et al., 2013).

Cellular-level disease tropism can reflect direct damage by the pathogen, for example due to cell rupture by lytic viruses, or to disrupted cellular physiology. However, cellular-level pathogen tropism and disease tropism are not necessarily identical. For example, Zika virus infects multiple cell types, including microglia-like cells, astrocytes and neural progenitor cells, but only caused significant death of the latter cell type (Muffat et al., 2018). Disease tropism may also be due to collateral damage from pathogen invasion or pathogen-mediated effects on adjacent cells. For example, in the absence of myeloperoxidase, superoxide production in *Salmonella*-infected neutrophils damages the surrounding cells (Schürmann et al., 2017). Co-culture of neurons with *T. cruzi* and IFNγ-activated macrophages led to neuronal death, which was abrogated by inhibitors of nitric oxide production (Almeida-Leite et al., 2007). Zika virus increases lipid droplet levels not only in infected cells but also in adjacent uninfected cells (Chen et al., 2020). Many pathogens also lead to immune cell exhaustion. Thus, *Mycobacterium tuberculosis* leads to CD8^+^ T cell exhaustion, even though it does not directly infect these cells (Russell et al., 2019).

### Tissue and Organ Tropism

Organ and tissue tropism are relevant to both intracellular and extracellular pathogens. Some pathogens may show uniform burden throughout a given organ [*e.g. Pseudomonas aeruginosa* in the gallbladder (Bachta et al., 2020)], while others may show preferential tropism to select organ regions [*e.g.* higher *T. cruzi* load at the heart base in strain CL Brener infection of C3H/HeJ mice (McCall et al., 2017); higher norovirus load in the distal small intestine in wild-type C57BL/6 mice (Grau et al., 2020)]. In the case of intracellular pathogens, cellular tropism will strongly influence tissue tropism, for example if cells expressing the necessary receptor are more common in a given organ or tissue. In the case of SARS-CoV-2, viral tropism to the respiratory and gastrointestinal tract has been linked to co-expression of the ACE2 receptor and TMPRSS2 protease in these tissues (Sungnak et al., 2020; Ziegler et al., 2020). Tropism to olfactory epithelium may be potentiated by its expression of neuropilin-1, which enables entry of SARS-CoV-2 with furin-cleaved spike protein (Cantuti-Castelvetri et al., 2020). Such determinants of tissue tropism should thus be apparent in cell culture models, where the pathogen can only infect cells from the target organ. For example, respiratory enterovirus strains are restricted to the lung *in vivo* and show much more restrictive *in vitro* tropism than disseminative enteric enterovirus strains (Royston et al., 2018). In contrast, many other intracellular pathogens are promiscuous in cell culture [*e.g. T. cruzi* (Franco et al., 2019) or *Toxoplasma gondii* (Cabral et al., 2016)]. *T. cruzi* shows initial broad tropism *in vivo* during acute infection, followed by more restricted pathogen and disease tropism in chronic disease stages [mainly heart, stomach and colon, with some exceptions (Lewis et al., 2014; Lewis et al., 2016; Hossain et al., 2020)]. In the case of *T. gondii*, broad spatial distribution can be observed following infection with a virulent bioluminescent strain, including localization to the intestines, lung, liver, brain, heart, and kidney (Saeij et al., 2005). However, fine study of infected cell types in the brain indicates that parasites preferentially infect neurons over astrocytes *in vivo*, and this preference is only partially abrogated by immunosuppression (Cabral et al., 2016)

Sites of initial pathogen entry, infection of highly migratory cells such as macrophages, or pathogen motility, will also determine whether a pathogen remains restricted to its initial site of invasion or can disseminate to other organs (see below). Pathogen and disease tropism are thus also influenced by circulatory patterns, leading for example to the accumulation of *Leishmania donovani-*infected macrophages (McCall et al., 2013) and *Plasmodium-*infected red blood cells (Brugat et al., 2014) in the spleen. Direct binding to microbiota bacteria may also facilitate viral invasion (Erickson et al., 2018).

Host metabolism and thus nutrient and immunomodulatory metabolite availability also differ strongly between organs (Quinn et al., 2020). For example, levels of purines, aspartate and histidine are lower in the skin than the liver (Murakami et al., 2014). Variable nutrient availability restricts the tropism of pathogens with strict nutritional requirements. Dermotropic *Leishmania* have higher transporter expression, which may enable them to address skin-associated nutrient limitations, in contrast to viscerotropic *Leishmania* (McCall et al., 2015). *Streptococcus pneumoniae* adaptations to ear vs. circulatory environments has been tied to enhanced ability of blood *S. pneumoniae* strains to utilize raffinose as a carbon source (Minhas et al., 2019). Ear-tropic and lung-tropic *S. pneumoniae* strains also express different nutrient transporters (Minhas et al., 2020). Nutrient competition is a key mechanism by which the microbiota restricts pathogen colonization (Lam and Monack, 2014; Brugiroux et al., 2016). Depletion of such commensals enhances the risk of colonization by pathogens such as *Clostridioides difficile* (Buffie et al., 2015). The balance between microbiota-derived butyrate and acetate also shapes colonization-associated gene expression in *Campylobacter jejuni* and may explain its preferential tropism for the colon over the small intestine (Luethy et al., 2017). Metabolites also regulate immune responses, influencing pathogen organ tropism. For example, the intersection between secondary bile acid production by the microbiota and host bile acid receptor FXR levels regulate cellular production of IFN-λ and consequently norovirus tropism in the intestines (Grau et al., 2020). Bile acids also regulate norovirus endocytosis and virus release from endosomes (Murakami et al., 2020).

Immune responses strongly regulate pathogen tropism, as evidenced by the many cases of atypical disease presentations in immunocompromised individuals: central nervous system rather than cardiac involvement in AIDS-Chagas disease patients (Pinazo et al., 2013), invasive fungal dermatophyte infections in primary immunodeficiency patients (Pilmis et al., 2016), or broad Human Cytomegalovirus (HCMV) tropism in systemic lupus erythematosus (SLE) patients receiving immunosuppressive therapy (Arai et al., 2012). In experimental models, immunosuppression abolishes the select chronic-stage tropism of pathogens such as *T. cruzi (* Lewis et al., 2016 *).* Some organs are less accessible to cells of the adaptive immune response; such immune privilege may explain *Trypanosoma brucei* (McCall and McKerrow, 2014) or *T. gondii* (Saeij et al., 2005) brain tropism. However, beyond the well-characterized case of immune privilege, immune responses can also differ between organs that are freely accessible to immune cells. For example, CD4^+^ T cells in the liver adopt an IL-10-producing phenotype during *Salmonella* Typhimurium infection, leading to M2 macrophage polarization and long-term bacterial persistence in the liver; in contrast, CD4^+^ T cells in the spleen produce high IFNγ and low IL-10, leading to bacterial clearance (Kurtz et al., 2020). Immune restriction of pathogen tropism is not limited to adaptive responses: for example, West Nile virus is restricted from the liver due to rapid induction of type I interferons (Suthar et al., 2013).

As with pathogen tropism, disease tropism may also occur throughout a given organ, or be restricted to select organ regions. For example, *T. cruzi* leads to pathognomonic apical cardiac aneurysms, even though cardiac and cardiac apex parasite load are low (Marin-Neto et al., 2007). Genital HSV-2 infection leads to gastrointestinal and urinary manifestations (Khoury-Hanold et al., 2016). Strikingly, in the case of HSV-1 experimental infection, while viral colonization was observed in the dorsal root ganglia and large intestine, tissue damage was strongest in the latter, driven by excessive neutrophil recruitment and destruction of intestinal ganglia (Khoury-Hanold et al., 2016). Disease tropism to select organs and tissues is influenced by preferential sites of pathogen tropism, the ability of different organs to function even in the presence of pathogens (tolerance) and repair ability after pathogen clearance (see below).

### Tropism Goes Global

Subcellular, cellular, tissue and organ tropism are most relevant to an individual’s experience of disease. However, pathogens and disease do not show equal distribution across the globe. Thus, geography strongly influences disease tropism, from a continental scale to differential disease incidence between neighborhoods within a given city.

On a broad geographic scale, external factors such as climate influence vector and vector-borne disease distribution [e.g. (Li et al., 2019)]. Likewise, changes in reservoir animal host tropism and reservoir behavior will influence the risk of human exposure (Zhang et al., 2018). Population movements shape global disease tropism, by introducing infected individuals in new locations (Alawieh et al., 2014). Large-scale population movements are often tied to political instability, which in turn influences the availability of disease control measures and population health (Du et al., 2018). Such factors may also play out on a smaller scale, within a city or even a building (Chng et al., 2020).

The microbiota is strongly influenced by these extrinsic factors: urbanization, loss of traditional foods and living practices, and exposure to processed food, consumer chemicals and antimicrobials (Sankaranarayanan et al., 2015; Vangay et al., 2018; McCall et al., 2020). In turn, microbiota composition affects susceptibility to infectious diseases [*e.g.* (Buffie et al., 2015; Villarino et al., 2016)]. Thus, the global variation in microbiota composition may be responsible for some of the observed global variations in infectious disease prevalence. In addition to this “second genome” (Grice and Segre, 2012), variations in genetic background between populations may also lead to variable pathogen and disease tropism between geographic regions, although this is challenging to deconvolute from healthcare and service access, behavioral patterns, vector and reservoir tropism. Well-characterized genetic factors that influence tropism in the context of malaria and show differential prevalence between geographic regions include Duffy receptor presence/absence vs. susceptibility to *P. vivax* malaria (Twohig et al., 2019).

Overall, pathogens and infectious diseases show preferential tropism at multiple levels, from the microscopic to the planetary scale. In the following sections, we will examine in more detail the specific factors that influence tropism.

## The Ordered Steps of Pathogen Tropism

### The Starting Line: Route of Infection

In the case of pathogens newly-introduced into a mammalian body, initial tropism will be determined by the route of administration: vector-borne transmission will lead to pathogen deposition in the skin and/or vasculature, while food-borne parasites will initially colonize the gastrointestinal tract, and so on. The initial method of entry can also shape final tropism, with transmission by the tick vector leading to broader tissue tropism for several *Borrelia burgdorferi sensu lato* complex strains than needle injection (Sertour et al., 2018). In the case of pathobionts, members of the microbiota that can be pathogenic under certain circumstances, initial tropism will depend on which regional microbiota they came from, and the factors that led to them becoming pathogenic, such as disruption of local mucosal surfaces leading to invasion [*e.g.* (Ayres et al., 2012)].

### Entering a Propitious Environment

Having entered the mammalian body, the ability of the pathogen to remain will be influenced by the availability of appropriate niches at the entry site. An intracellular pathogen that does not encounter the necessary invasion receptors at the site of colonization and is unable to disseminate will lead to an abortive infection. pH, temperature and nutritional requirements also determine whether the invading pathogen can establish itself. For example, MERS is resistant to fed-state simulated gastric fluid and partially resistant to fed-state simulated intestinal fluid; however, fasted-state simulated gastric fluid kills the virus. Thus, timing of viral exposure vs. meals may influence the success of oral MERS infection (Zhou et al., 2017). Attachment is also relevant to extracellular pathogens; for example, enteroaggregative *E. coli* showed differential patterns of adhesion on enteroids from each intestinal segment (Rajan et al., 2018). Temperature effects may be direct, due to differential pathogen proliferation at temperatures associated with visceral organs vs. skin or mucosal surfaces (McCall and Matlashewski, 2010; McCall and Matlashewski, 2012), or indirect *via* effects on the host. For example, airway epithelial cells produce more interferon at 37°C than 33°C, thus restricting rhinovirus to the cooler upper airways (Foxman et al., 2015).

Host- or microbiota-derived metabolites can either promote pathogen persistence (production of a key nutrient) or inhibit it (lack of a key nutrient; production of toxic host metabolites). As an example of the former mechanism, gastrointestinal *Salmonella* retain genes that enable utilization of alternative carbon sources, compared to extraintestinal *Salmonella* which are not able to access these resources. Extraintestinal strains also show loss of functions associated with anaerobic metabolism (Nuccio and Bäumler, 2014). For the latter mechanism, high levels of D-serine in the bladder prevent enterohemorrhagic *E. coli* (EHEC) bladder colonization by reducing type 3 secretion system virulence factor expression, host cell attachment and pedestal formation (Connolly et al., 2015). The microbiota can also directly interfere with or promote invasion by the pathogen. For example, successful influenza A virus transmission is inhibited by nasal carriage of *Streptococcus pneumoniae*, which cleaves host nasal sialic acid, the receptor for influenza (Ortigoza et al., 2018).

Lastly, colonization will also be determined by whether a pathogen can withstand or co-opt the initial immune responses. For example, *Leishmania* parasites infect neutrophils recruited to the initial site of the sandfly vector bite (Peters et al., 2008). In contrast, presence of influenza-specific resident memory T cells in the upper respiratory tract protected against nasal colonization and viral dissemination to the lung (Pizzolla et al., 2017).

Host regulation of pathogen tropism can even apply to prions (PrP^Sc^): PrP^Sc^ strain differential tropism is related to the expression levels of endogenous healthy PrP^C^ proteins (Béringue et al., 2020). Initial steps of pathogen tropism can already be active processes. For example, pathogen proteins will subvert host cell functions to determine subcellular localization. Indeed, the *Shigella* type III secretion system translocon is sufficient to induce cytoplasmic localization when expressed ectopically in *Escherichia coli* (Du et al., 2016).

### Leaving the Initial Site of Colonization

After setting up a “beachhead” at the site of initial invasion, pathogens will stay at that site or disseminate. Local persistence vs. dissemination is determined by the site of initial colonization, obstacles encountered during dissemination, and whether other locations are suitable for pathogen persistence and proliferation. Indeed, simian immunodeficiency virus (SIV) disseminates from the female genital tract into the bloodstream only when local viral loads exceed a certain threshold (Feder et al., 2017). Depending on whether fibroblasts or endothelial cells are infected with HCMV, released viral progeny is either tropic to both cell types, or restricted to fibroblasts (Scrivano et al., 2011). A similar process is observed in EBV infection, where viral progeny from B cells is better at infecting epithelial cells than B cells, and vice versa (Borza and Hutt-Fletcher, 2002). The quality and strength of local immune also determines whether systemic dissemination can occur. For example, stimulating innate immune responses in the skin prevented arboviral dissemination (Bryden et al., 2020). Conversely, immune responses can promote dissemination: for example, varicella zoster virus infection of tonsillar T cells promoted trafficking to tissues (Sen et al., 2014).

The ability to leave the site of colonization is also linked to pathogen abilities to actively penetrate tissues, for example *via* induction of parasite and host proteases in *Entamoeba histolytica* infection, enabling invasion out of the gastrointestinal tract (Thibeaux et al., 2014; Siqueira-Neto et al., 2018). Zika virus also co-opts host proteases to cross the blood-testis barrier (Hui et al., 2020). *S. aureus* may invade bones *via* forces generated by cell division (Masters et al., 2020). Inflammasome induction by *Plasmodium falciparum* histidine-rich protein II leads to loss of blood-brain barrier integrity (Pal et al., 2016) and influenza H5N1 infection of endothelial cells promotes vascular leakage and dissemination to extrapulmonary sites (Tundup et al., 2017). Invasiveness does not have to be constitutive: for example, *Shigella* turn on type 3 secretion system (T3SS) needle production in anaerobic environments such as the gut lumen; approaching mucosal tissues leads to induction of effector secretion (Marteyn et al., 2010).

Such active penetration may not be necessary if the host presents with pre-existing damage. For example, loosened intestinal barrier in hyperglycemic mice increased ability of *Citrobacter rodentium* to colonize spleen and liver (Thaiss et al., 2018). Similarly, immune-mediated disruptions to lung blood vessels during lung transplant led to increased iron availability that promoted *Aspergillus fumigatus* invasiveness (Hsu et al., 2018). Dissemination and invasiveness is also facilitated by pathogen motility [*e.g.* bacterial flagella (Cullender et al., 2013)], access to the lymphatic system [*e.g.* group A Streptococci, mediated by capsule hyaluronan interaction with lymphatic vessel endothelial receptor 1 (Lynskey et al., 2015)], infection of mobile host cells [*e.g. Chlamydia trachomatis* infection of dendritic cells (Howe et al., 2019)] or by taking advantage of cell-cell connections [*e.g.* HSV-1 dissemination from initial sites of vaginal colonization to the dorsal root ganglia, then spine, and then colon enteric nervous system *via* peripheral nociceptors (Khoury-Hanold et al., 2016)].

One caveat is that it is often challenging to differentiate between a pathogen that remains at local sites of initial invasion without disseminating to other tissues, and a pathogen that does disseminate but is unable to establish itself anywhere but at the initial site. As an example, we observed high *T. cruzi* load at the heart base during acute experimental *T. cruzi* infection (McCall et al., 2017); is this due to a lack of parasite dissemination beyond this cardiac region, or to rapid parasite killing at the heart apex? The observation of higher antiparasitic IFNγ at the heart apex argues for the latter scenario (McCall et al., 2017). Conversely, phagocytic cells infected with *L. major* parasites are less likely to migrate out of the site of intradermal infections than *L. donovani-*infected cells, indicating that the dermotropism of *L. major* is due to the first scenario (Zhang et al., 2003). Many immune mechanisms are designed to capture and kill pathogens during the dissemination process. As an example, liver Kupffer cells phagocytose circulating pathogens (McDonald et al., 2020) and circulating antibodies neutralize key pathogen surface molecules or target them for degradation (Engstler et al., 2007). These mechanisms can be subverted by pathogens adapted to live in macrophages such as *Leishmania (* Beattie et al., 2013 *)*, by loss of long-range interactions between gut microbiota and immune function during dysbiosis (McDonald et al., 2020), or by pathogen surface antigenic variation (Engstler et al., 2007).

High temporal and spatial resolution series of luminescent animal models may conclusively resolve this issue (see below). Importantly, disease resolution or lack of apparent disease at any time at the site of initial pathogen invasion does not mean lack of low-level pathogen persistence. For example, *T. cruzi* persist at low levels in the skin in mammalian models, facilitating pathogen transmission, in the absence of visible skin lesions (Ward et al., 2020).

### Colonizing and Persisting at New Sites

Colonizing additional sites is influenced by many of the same factors that influence initial colonization: nutrient and host cell availability (McCall et al., 2015; Quinn et al., 2020; Sungnak et al., 2020; Ziegler et al., 2020), competition (Lam and Monack, 2014), thermal and stress tolerance (Scull et al., 2009; McCall and Matlashewski, 2010; McCall and Matlashewski, 2012), surviving immune responses, for example through colonization of immune privileged organs (see above, as for *T. gondii* or *T. brucei*) (Saeij et al., 2005; McCall and McKerrow, 2014) or co-opting defense pathways [*e.g.* (Mandal et al., 2017; Howe et al., 2019; Ziegler et al., 2020)]. For the multiple pathogens that initially show broad tissue tropism followed by more restrictive tropism [*e.g. T. cruzi, T. gondii* (Saeij et al., 2005; Lewis et al., 2014)], basal nutrient availability may be less restrictive; in contrast, tissue metabolic remodeling or induction of immunomodulatory metabolites may be more important. For example, we observed infection-induced increases of the metabolite kynurenine mainly in the large intestine and to a lesser extent in the stomach following *T. cruzi* infection. Kynurenine induces regulatory T cells and may thus contribute to parasite persistence in the colon (Hossain et al., 2020).

### Determinants of Disease Tropism

Disease tolerance is the ability to withstand the deleterious effects of infection (McCarville and Ayres, 2018). Resilience is the ability to return to health following the clearance of a pathogenic insult (Torres et al., 2016). While this has been predominantly studied at the level of the whole organism, our findings of localized metabolic perturbations and localized metabolic restoration by pharmacological interventions with constant parasite burden argue for spatial disease tolerance (Hossain et al., 2020). Thus, we find that the heart apex appears to be less tolerant to acute *T. cruzi* infection than the heart base (McCall et al., 2017), while select intestinal regions have a higher capacity to return to normal metabolism in the chronic stage of experimental infection (Hossain et al., 2020).

Some of these characteristics are intrinsic to a tissue, for example with regards to responses to initial infection (Rajan et al., 2020), or ability to regenerate; thus, some locations are more prone to persistent infectious disease damage than others. Cardiac damage, for example, is challenging to reverse, even after the pathogen is cleared [*e.g.* (Morillo et al., 2015)]. However, the location of damage (disease tropism) is naturally also influenced by pathogen localization and its intersection with organ, tissue and cellular characteristics. For example, lung infection with SARS-CoV-2 leads to lung lesions and respiratory impairment, related to the high viral receptor expression in lung pneumocytes (Finch et al., 2020; Ziegler et al., 2020). These lesions are highly localized; however, virus can also be detected in the kidney, liver, heart, and periodically in the brain (Puelles et al., 2020). SARS-CoV-2 infection is associated with elevated interferon production, but only in the lower respiratory airways; IFNλ impairs the lung barrier and may thus promote increased disease severity and superinfection *via* those sites (Broggi et al., 2020). This differential interferon production may be related to the temperature sensitivity of the interferon response reported in rhinovirus infection (Foxman et al., 2015). Neurological symptoms of COVID-19 have been particularly mysterious. Recent work demonstrating SARS-CoV-2 tropism to choroid plexus cells and causing breakdown of the blood-central nervous system barrier integrity (Pellegrini et al., 2020), illustrates how pathogen tropism informs the study of disease tropism.

The ability of a pathogen to cause collateral damage to uninfected cells, tissues and organs will also determine disease tropism. This damage may be due to mediators produced by the pathogen [*e.g.* (Thibeaux et al., 2014)], the activation of non-specific immune responses leading to damage of adjacent cells [*e.g.* (Almeida-Leite et al., 2007; Schürmann et al., 2017)], or compensatory mechanisms in response to direct tissue damage. An example of the latter mechanism would be hypertrophy of surviving cardiomyocytes in chronic *T. cruzi* infection, as part of an effort to maintain heart function (Garg et al., 2003). Although collateral damage is assumed to neighbor pathogen localization, this may not always be true. For example, in an immunocompetent mouse model of Zika virus infection, fetal abnormalities were observed even in animals where no placental or embryonic viral RNA were detected, caused by type I interferon-mediated placental damage (Szaba et al., 2018).

## Methods to Study Tropism

Locating pathogens can be performed in animal models by visual observation or microscopy [e.g. (Peters et al., 2008; Miner et al., 2016; Stieh et al., 2016)], bioluminescence [e.g. (Lewis et al., 2014; Lewis et al., 2016)], flow cytometry/FACS [e.g. (Lee et al., 2017)], PCR [e.g. (Stieh et al., 2016)] or single-genome sequencing [e.g. (Feder et al., 2017)]. Timecourse analyses are essential to determine the sequence of colonization events that determine tropism. Fluorochrome switching or photoconversion track the history of a given pathogen, by monitoring which locations were traversed (Müller et al., 2013; Tan et al., 2016). Such techniques must differentiate true tissue-tropic pathogens from pathogens that are at high levels in the circulation, including the organ microvasculature, for example by perfusion prior to organ imaging (Lewis et al., 2014).

Animal models may not always fully reflect human disease and pathogen tropism, but facilitate systematic tissue access, invasive and timecourse sampling, with fewer behavioral confounders. Humanized mouse models offer an attractive, albeit still expensive, alternative (Wahl et al., 2019). In humans, pathogen location can be determined non-invasively using multiple clinical specimens to broadly define tropism (Rodrigues-Dos-Santos et al., 2018; Parasa et al., 2020). Complementary methods include endoscopy and medical imaging (Werneck-Silva and Prado, 2009; Godet et al., 2016). Lastly, autopsies can determine pathogen tropism in humans, in fatal infections (Balsitis et al., 2009). Disease tropism can likewise be established by visual assessment of microscopic and macroscopic damage [*e.g.* (Edler et al., 2020)], or expression levels of a specific damage marker [*e.g.* (Varga et al., 2020)].

However, none of these approaches tell us *why* pathogen or disease tropism occurs at those select sites. To identify determinants of tropism, additional approaches are required. Transcriptomic analyses can identify viral receptors, for example by comparing transcripts encoding membrane-associated proteins between cells susceptible and resistant to viruses (Karakus et al., 2019). Single-cell sequencing can determine the specific cellular subsets that express the key viral receptors and the factors regulating their abundance, as was recently performed to identify target cells of SARS-CoV-2 (Ziegler et al., 2020). CRISPR and RNAi systematic screens *in vitro* identify key receptors for pathogen entry [*e.g.* (Karakus et al., 2019)] and metabolic pathways essential for intracellular pathogen proliferation [*e.g.* (Caradonna et al., 2013)]. Infection with a pool of RNAi and miRNA viruses defined host factors that restrict Sindbis and influenza A virus infection *in vivo*, in the spleen and lung, respectively (Varble et al., 2013; Benitez et al., 2015). Retrieving virus from multiple tissues would expand this method to study factors driving differential tissue tropism. Characterizing pathogen and host gene expression can also determine sites of less-productive pathogen proliferation and host pathways associated with tissue damage, as was performed in HSV-1 infection (Khoury-Hanold et al., 2016). Metabolic models for different cell types or different organs (Noronha et al., 2018; Thiele et al., 2020) can identify which sites are candidates for pathogen proliferation, complemented by comparative metabolomic analyses of multiple organs and tissue regions (McCall et al., 2017; Hossain et al., 2020; Quinn et al., 2020). We recently demonstrated that sites of highest metabolic perturbation in the gastrointestinal tract and the heart following experimental *T. cruzi* infection match known macroscopic sites of *T. cruzi-*induced damage in patients, providing a novel way to determine disease tropism (Dean et al., 2020; Hossain et al., 2020).

Identifying additional host and pathogen genetic factors that drive tropism can be performed by experimental infections in various host-pathogen strain combinations [*e.g.* (Lewis et al., 2016; Minhas et al., 2019)]. Genomic, transcriptomic and proteomic comparison of related pathogen strains can provide insight into the factors controlling tropism, for example in the context of *Leishmania* parasites (Zhang and Matlashewski, 2010; Zhang et al., 2014). *In vivo* selection promoting switch from one site of tropism to another can be particularly helpful in this context, because parental and selected strain are closely related genetically (McCall et al., 2015; Lypaczewski et al., 2018). Genetically modified pathogen strains are essential in this context. One clever example of genetic manipulation introduced cell-type-specific microRNA target sites into the influenza H5N1 genome, thus generating viral strains unable to infect specific cell types *in vivo* and demonstrating a requirement for endothelial cell infection in vascular leakage, inflammation, and viral dissemination to extrapulmonary sites (Tundup et al., 2017).

Validating candidate determinants of tropism is essential, *via* knockout/knockdowns [*e.g.* (Zhang and Matlashewski, 1997)], antibody-based blocking or depletion [*e.g.* (Guzzo et al., 2017)], ectopic expression of tropism-associated proteins in resistant cell types [*e.g.* (Zhang et al., 2008; Zhang et al., 2014)], or pharmacological modulation [*e.g.* (Thaiss et al., 2018; Howe et al., 2019; Bryden et al., 2020; Grau et al., 2020)]. Mendelian randomization may show utility in select cases to validate host determinants in humans (Reilly et al., 2018), but may only be sufficiently powered for very prevalent pathogens.

## Translational Implications

Beyond fundamental knowledge into pathogenic processes, understanding tropism can determine modes of transmission and thus intervention. For example, detecting Zika virus in the genital tract provides a mechanism to support sexual transmission of the virus and interventions to prevent this transmission route (Ma et al., 2016). Understanding tropism in COVID-19 could determine methods to prevent transmission, for example by demonstrating whether fecal transmission should be a concern (Xiao et al., 2020), but also patient staging and monitoring priorities (Mao et al., 2020), explain atypical disease presentations (Varga et al., 2020), and develop new treatment approaches. Infectious disease drug discovery focuses predominantly on decreasing or eliminating pathogen load. However, newer anti-virulence treatment strategies, for example type III secretion system inhibitors (Veenendaal et al., 2009), may impact tropism given the role of type III secretion in *Shigella* tropism [see above, (Du et al., 2016)], and thus lead to new tropism-modulating therapeutics.

However, tropism should also be a consideration even for antimicrobials that do not directly aim to affect it. Indeed, certain sites may be less accessible to administered therapeutics, leading to treatment failure and increased risk of antimicrobial resistance. Such failures may be an intrinsic property of a drug being unevenly distributed to different organs, with poorer distribution to the major sites of pathogen tropism. For example, AmBisome, while effective against *L. donovani*, was ineffective at curing *T. cruzi* infections. AmBisome accumulates in the liver, spleen and lungs; liver and spleen are major sites of *L. donovani* tropism but not of *T. cruzi* (Sundar et al., 2010; Cencig et al., 2011; McCall et al., 2013; Lewis et al., 2014). The tissue environment, including nutrient or oxygen availability, will influence pathogen metabolism, and antimicrobial agent efficacy is often reliant on pathogen metabolic activity (Stokes et al., 2019; Kowalski et al., 2020). Likewise, changes in the metabolic environment induced by antimicrobial treatment will affect host immune responses and pathogen clearance (Yang et al., 2017), mechanisms that are particularly important in the context of “static” rather than “cidal” treatments. Pathogen strains may also differ between tissue locations. In this context, the risk of selection of HIV tropic for CXCR4 co-receptor-expressing rather than CCR5-expressing cells was a major concern in the development of CCR5 antagonists (Cascajero et al., 2018). In parallel, cofactor or virulence-associated protein expression, whether host or pathogen-derived, may differ between cell types and tissue locations. As an example, hydroxychloroquine failure in SARS-CoV-2 clinical trials in comparison to early *in vitro* successes may be attributed to initial studies using Vero cells for compound activity testing. Vero cells are not major natural sites of SARS-CoV-2 tropism; unlike lung cells, viral entry into Vero cells relies on endosomal cathepsin L rather than TMPRSS2 (Hoffmann et al., 2020). Considerations of cellular tropism are thus essential when designing an appropriate *in vitro* drug development or high-throughput screening assay.

Distinguishing between spatial treatment failure due to unequal drug penetration and treatment failure due to pathogen resistance or tolerance to drug effects necessitates a combination of approaches, including improving spatial and temporal resolution of classical pharmacokinetic/pharmacodynamic (PK/PD) studies, spatial characterization of pathogen gene expression in response to treatment and pathogen isolation followed by *in vitro* antimicrobial susceptibility testing. Indeed, timing of treatment prior to Marburg virus testes colonization prevented viral persistence (Coffin et al., 2018). Although cecal and cervical azithromycin levels were comparable, *C. trachomatis* were less readily cleared from the cecum (Yeruva et al., 2013). Importantly, although PK/PD studies are usually performed in healthy animals, there is a need to expand these studies to the context of infection, where select disease tropism may alter drug distribution and clearance (Hoffman et al., 2020). Tropism considerations may thus be a major reason for the failure *in vivo* of new chemical entities that were promising *in vitro.*

Immunomodulators are being considered to mitigate infection-induced damage, for example in COVID-19 (Ingraham et al., 2020). However, local and peripheral immune responses may differ significantly; for example, different patterns of interferon expression were observed in peripheral blood mononuclear cells vs. lung cells in bronchoalveolar lavage fluid of COVID-19 patients (Overholt et al., 2020), and in upper vs. lower airways (Broggi et al., 2020). Regulatory T cell depletion also had opposite effects on murine cytomegalovirus (MCMV) reactivation in the spleen and salivary gland (Almanan et al., 2017). These observations highlight the need to understand tropism before developing immunomodulatory interventions. Likewise, vaccine research most often relies on assessment of peripheral immune responses; these examples of differences between localized and systemic immune responses highlight the need for increased characterization of tissue-specific immune responses in studies of vaccine mechanism of protection.

Lastly, tropism is also relevant to diagnostic test development: methods that rely on biofluid monitoring may not detect pathogens with specific tissue tropism. Indeed, this is one of the causes of the high rates of false negatives in PCR-based diagnosis of chronic Chagas disease (Murcia et al., 2010; Pérez-Ayala et al., 2011). Likewise, disease-staging and prognostic approaches that only rely on circulating biomarkers without considering the damage occurring at sites of pathogen tropism may be less successful than methods that consider damage pathways, and then search for those specific markers in accessible biofluids (McCall et al., 2017). Thus, considerations of pathogen and disease tropism should be at the heart of translational infectious disease research.

## Conclusions and Unresolved Questions

Overall, a tropism perspective on pathogenesis can help understand why disease happens and how to intervene. However, comprehensive studies on tropism are often lacking; most focus on a few subcellular locations, cell types, tissues, or organs. Characterization of tropism within an organ, rather than just between organs is also necessary. Successful integration of multiple layers of information, from histology to ‘omics and pharmacological validation will enrich research in tropism. Given the key role of small molecules as microbial building blocks, regulators of pathogen entry, and immunomodulators, metabolomics should be prominently used in such studies. Tropism in the context of co-infection and co-morbidities has rarely been investigated. Lastly, considerations of the translational aspects of pathogen and disease tropism are still under-explored; however, this will be extremely valuable to guide drug and biomarker development.

## Author Contributions

The author confirms being the sole contributor of this work and has approved it for publication.

## Funding

Research on tropism and translational applications in the McCall laboratory at the University of Oklahoma is or was supported by NIH award number 1R21AI148886, PhRMA foundation award number 45188 and a pilot grant from the Oklahoma Center for Respiratory and Infectious Diseases, funded by the National Institute of General Medical Sciences of the National Institutes of Health under Award Number P20GM103648.

## Conflict of Interest

The author declares that the research was conducted in the absence of any commercial or financial relationships that could be construed as a potential conflict of interest.
